# Molecular Epidemiology of *Escherichia coli* Resistant to Carbapenems, Fluoroquinolones, and Aminoglycosides Isolated from One of the Largest Hospitals in Vietnam in 2014–2019

**DOI:** 10.1155/2024/2711353

**Published:** 2024-01-31

**Authors:** Tohru Miyoshi-Akiyama, Do Van Thanh, Truong Thai Phuong, Nguyen Quang Huy, Pham Thi Phuong Thuy, Teruo Kirikae, Pham Hong Nhung, Norio Ohmagari

**Affiliations:** ^1^Pathogenic Microbe Laboratory, Research Institute, National Center for Global Health and Medicine, Toyama 1-21-1, Shinjuku-ku, Tokyo 162-8655, Japan; ^2^Bach Mai Hospital, 78 Gia Phong Road, Phuong Mai, Dong Da District, Hanoi, Vietnam; ^3^NCGM-Bach Mai Hospital Medical Collaboration Center, 78 Gia Phong Road, Phuong Mai, Dong Da District, Hanoi, Vietnam; ^4^Department of Infectious Diseases, National Center for Global Health and Medicine, Toyama 1-21-1, Shinjuku-ku, Tokyo 162-8655, Japan; ^5^Department of Microbiology, Juntendo University School of Medicine, 2-1-1 Hongo, Bunkyo-ku 113-0033, Japan; ^6^Department of Microbiology, Hanoi Medical University, 1 Ton That Tung, Dong, Da District, Hanoi, Vietnam; ^7^Disease Control and Prevention Center, National Center for Global Health and Medicine, Toyama 1-21-1, Shinjuku-ku, Tokyo 162-8655, Japan

## Abstract

**Introduction:**

Multidrug-resistant (MDR) Gram-negative bacilli including carbapenem-resistant Gram-negative *Enterobacteriaceae* (CRE) threaten global health. Little is known, however, about the distribution of antimicrobial resistance genes in MDR isolated from patients in Vietnamese hospitals. In this study, we collected MDR *Escherichia coli*, defined as E. coli resistance against all fluoroquinolones, aminoglycosides, and carbapenems.

**Aim:**

This study was designed to clarify the molecular epidemiology of *Escherichia coli* isolates resistant to carbapenems, fluoroquinolones, and aminoglycosides isolated from patients admitted to one of the largest hospitals in Vietnam in 2014–2019 based on both whole-genome sequencing (WGS) and phenotypic data. *Methodology*. Sixty-seven Vietnamese isolates screened by drug resistance by the disk test were subjected to WGS, and their sequences were analyzed to determine their multilocus sequence type (MLST), O-types, H-types, distribution of drug resistance genes, plasmid types, pathogenicity islands (PIs), virulence factor distribution, and phylogenetic evolution using the WGS data.

**Results:**

Among the STs detected, ST410 was relatively dominant. Dominant O-types and H-types were O102 and H9 and showed some links, such as those between O102 and H8. The most dominant plasmid type and carbapenemase type were 4 and NDM-5, respectively. MLST, O-types, H-types, plasmid types, and types of carbapenemases were very heterogeneous among the isolates, with no clear correlation between them. Dominant plasmid type carrying drug resistance gene was IncQ1_1. The percentage of isolates positive for drug resistance genes, such as anti-beta-lactams and aminoglycosides, was relatively high because the isolates screened were resistant to carbapenems, fluoroquinolones, and aminoglycosides.

**Conclusions:**

MDR *E. coli* isolates isolated at a high-volume Vietnamese hospital were very heterogeneous, suggesting that they were acquired from different sources, including nosocomial infection, animals, and water. Eradication of MDR *E. coli* from hospitals and other clinical environments is very challenging because a single measure may be ineffective.

## 1. Introduction

Multidrug-resistant (MDR) Gram-negative bacilli including carbapenem-resistant Gram-negative *Enterobacteriaceae* (CRE) threaten global health. Although *Enterobacteriaceae* including *E. coli* showing MDR phenotype have been detected in Southeast Asia, most studies in Vietnam have focused on *Klebsiella pneumoniae* [1]. Little is known about the distribution of antimicrobial resistance (AMR) genes in MDR *E. coli* isolated from clinical settings in Vietnam and the correlation between these genes and MDR phenotypes. To date, drug resistance genes have been analyzed in *E. coli* isolated from raw meat and shellfish [2], retail meats [3, 4] and shrimp [5], hospital wastewater [6], backyard chicken farms [7], fecal sludge and soil [8], retail chicken carcasses [9], young dairy calves [10], urban rodents [5], and fish gut contents [11].

The present study, a joint collaboration between Japan and Vietnam, was designed to clarify the molecular epidemiology of *E. coli* isolates resistant to carbapenems, fluoroquinolones, and aminoglycosides isolated from patients admitted to one of the largest hospitals in Vietnam in 2014–2019. We chose three classes of the antibiotics because if all of the three drugs classes are ineffective, it will be very difficult to treat the patients. The molecular epidemiology of these isolates was evaluated by whole-genome sequencing (WGS) and phenotypic analysis. Factors analyzed in these isolates included multilocus sequence types (MLST), O-types, H-types, distribution of drug resistance genes, plasmid types, pathogenicity associated islands (PIs), virulence factor distribution, and phylogenetic evolution.

## 2. Materials and Methods

### 2.1. *E. coli* Isolates in This Study

**Figure 1:**
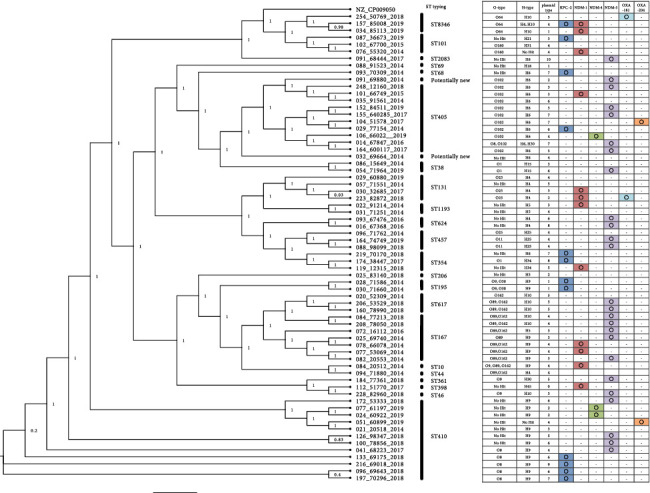
Phylogenetic analysis of all isolates from Vietnam, including typing results and the distribution of carbapenemase genes. ST, O-type, H-type, plasmid types, and the distribution of carbapenemase genes in all 67 *E. coli* isolates are presented. Phylogenetic analysis was performed with ParSNP 15, using *E. coli* NCCP15648 (NZ_CP009050) as the reference isolate.

Definition of MDR is described in GARDP (https://revive.gardp.org/resource/multidrug-resistant-mdr/?cf=encyclopaedia). In this study, we collected MDR *Escherichia coli*, which met the definition of MDR by Ministry of Health, Labour, and Welfare of Japan [12] showing resistance against all of fluoroquinolones, aminoglycosides, and carbapenems. To assess the epidemiology of MDR *E. coli* in clinical setting of Vietnam, MDR Gram-negative rods collected from patients at Bach Mai Hospital, Hanoi, Vietnam, in 2014–2019 were analyzed if they displayed resistance to imipenem, meropenem, amikacin, arbekacin, and ciprofloxacin, as shown by Kirby–Bauer (KB) disk (Eiken Chemical, Japan) methods. Because of ethical reasons, isolate information was separated from patient information such as department where patients were admitted and isolation source including blood, stool, or others. Of the 1133 MDR Gram-negative rod isolates collected, the number of MDR *E. coli* isolates was 67 (Figure 1). For ethical reasons, patients' background information was not available. Final confirmation of the identification of *E. coli* was performed by analyses of 16S rRNA sequence based on whole-genome sequence data.

### 2.2. Whole-Genome Sequencing

Genomic DNA was purified from the isolates using DNeasy Blood and Tissue kits (QIAGEN). Sequencing libraries were prepared using Nextera XT DNA Library Prep kits (Illumina), yielding pair end reads of 301 bp, or NEBNext Ultra II FS DNA Library Prep Kits for Illumina (NEB), followed by sequencing using MiSeq or HiSeq X (Illumina) yielding pair end reads of 301 bp or 150 bp, respectively, according to the manufacturers' instructions. Each isolate yielded approximately 700,000 to 8,000,000 reads. The resulting sequencing data were registered with the DNA Data Bank of Japan (DDBJ; accession no. DRA012227).

### 2.3. Bioinformatics

After trimming based on base quality (quality score limit = 0.05, removing reads with more than two ambiguous nucleotides and those less than 15 bp in length), the reads were *de novo* assembled to construct contigs without annotation using CLC genomics workbench 11 commercial software. Drug resistance genes were analyzed using the CLC genomics workbench microbial genomics module (detection parameters: 99% identity and 50% length coverage with manual correction), PlasmidFinder 2.1 (https://cge.cbs.dtu.dk/services/PlasmidFinder/) (95% identity and 90% length coverage with manual correction), VFDB (https://www.mgc.ac.cn/VFs/) (90% identity and 50% length coverage with manual correction) (https://cge.cbs.dtu.dk/services/), and PAIDB (https://www.paidb.re.kr/about_paidb.php) (90% identity and 50% length coverage with manual correction). Multilocus sequence type (MLST) was determined using the CLC genomics workbench microbial genomics module. O-type and H-type were analyzed by SeroTypeFinder (https://www.genomicepidemiology.org/services/). Phylogenetic analysis was performed with ParSNP [13] using *E. coli* NCCP15648 (NZ_CP009050) as the reference.

## 3. Results

### 3.1. *E. coli* Isolates Collected in This Study

From 2014 to 2019, 67 MDR *E. coli* isolates were collected at Bach Mai Hospital in Hanoi, Vietnam (Figure 1). Percentage of *E. coli* in the Gram-negative bacilli collected during study is presented in Table 1. During the study, we realized that *Acinetobacter baumannii* is quite dominant in our collection criteria. Thus, we presented two types of percentage in Table 1.

### 3.2. Relationships between the Isolates by Phylogenetic Analysis, Typing, and the Presence of Carbapenemase Genes

To analyze the relationship between the isolates, by phylogenetic analysis, we selected the carbapenemase genes as the drug resistance determinant in Figure 1. Phylogenic analysis using SNP concatemers showed that clustering of the isolates was essentially based on their sequence types (STs). These STs varied. Among the STs detected, ST410 was relatively dominant. Dominant O-types and H-types were O102 (10 isolates) and H9 (18 isolates) and showed some links, such as those between O102 and H8. The most dominant plasmid type and carbapenemase type were 4 and NDM-5, respectively. Analysis of O-types and H-types showed some links, such as those between O102 and H8. Further analysis showed that STs, O-types, H-types, and plasmid types were not clearly associated with the distribution of carbapenemase genes. Carbapenemase genes harbored by these isolates included KPC-2, NDM-1, NDM-4, NDM-5, OXA-181, and OXA-204. Among the carbapenemase genes detected, NDM-5 was most dominant (25 isolates). In 10 of the 67 isolates, however, known carbapenemase genes could not be detected. When we looked at the carbapenemases only, there might be some clusters. However, O-typing and H-typing results indicated that the isolates were not homogeneous. Because plasmid types are different in the possible cluster carrying, e.g., NDM-5, it is difficult to conclude that the isolates have different genetic background but carrying similar plasmid. There is a possibility that the carbapenemase genes of the 13 isolates were located on some mobile elements and lost during the re-cultivation. We also analyzed the presence of AcrAB-TolC efflux pump belonging to RND superfamily [14] using the sequences in *E. coli* K12 MG1655 as the queries and found that all of the isolates harbored the genes (data not shown). Thus, the efflux pump might contribute to the carbapenem resistance in the isolates.

### 3.3. The Distribution of Drug Resistance Genes

**Figure 2:**
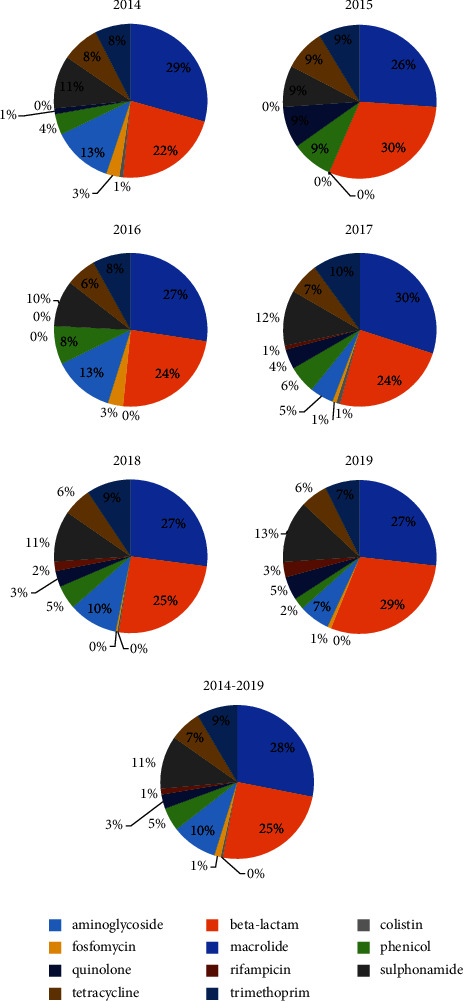
The relative percentage of genes conferring resistance to different classes of antibiotics during each year from 2014 to 2019 and in total. The genes conferring resistance to different class of antibiotics (aminoglycoside, beta-lactam, colistin, fosfomycin, macrolide, phenicol, quinolone, rifampicin, sulphonamide, tetracycline, and trimethoprim) are summarized in Supplementary Table 1. The number of the genes for each class was aggregated, and the percentage of genes for each class in the total genes was presented. Because only two MDR *E. coli* isolates were collected in 2015, some types of drug resistance genes, such as those conferring resistance to fosfomycin and macrolides, were not detected. The order of the drug resistance gene classes was in accordance with colors from left to right.

We used to screen MDR *E. coli* with 3 classes of antibiotics as described in Materials and Methods. Thus, the isolates collected were biased about the antibiotic susceptibility. But we thought that we should address the distribution of the drug resistance genes to ask whether they are homogeneous or not in the isolates collected. The distribution of drug resistance genes in these isolates was also analyzed (Supplementary Table S1 and Figure 2). Because these isolates were screened for resistance to three types of antibiotics, carbapenems, fluoroquinolones, and aminoglycosides, their proportion of drug resistance genes against beta-lactams and aminoglycosides was relatively high. During the year 2015, only two MDR *E. coli* isolates were collected. Thus, some types of drug resistance genes, such as those conferring resistance to fosfomycin and macrolides, were not detected (Figure 2). From 2014 to 2019, the percentage of isolates containing genes conferring resistance to fosfomycin decreased, while the percentage containing genes conferring resistance to rifampicin increased. Colistin resistance genes were detected in isolates isolated during 2016 and 2017. Although the relative percentage of the genes detected varied, no specific gene was associated with MDR activity (Supplementary Table 1), suggesting that these isolates were highly heterogeneous.

### 3.4. Analysis of the Pathogenicity Islands (PAIs) and Virulence Factor Genes

Analysis of PAI types among the isolates showed that seven types of PAI were present, with the two most abundant types being 14_ETT2 and 16_not_named (this name of PAI type was reported by PAIDB (http://www.paidb.re.kr/about_paidb.php))., which encoded a type III secretion system and a transketolase, respectively (Table S2). PIs associated with a type IV secretion system (04_HPI) and an effector (20_OI_57) were also relatively abundant. Analysis of the isolates showed the presence of 359 virulence factor genes. The proportion of MDR *E. coli* isolates harboring genes associated with enterobactin, type III secretion systems, and fimbriae was relatively high (Table S3), suggesting that some of the virulence factors detected in the isolates were associated with PAIs.

### 3.5. Relationship between Plasmids and Drug Resistance Genes

We analyzed the relationship of plasmids carried by the isolates and drug resistance genes carried by the plasmids by searching contigs carrying plasmid genes and then checking if the contig contained drug resistance genes or not. Of 323 contigs detected (data not shown), 17 contigs in 15 isolates carried at least one drug resistance gene (data not shown). Dominant plasmid gene in contigs carrying drug genes was IncQ1_1 (7 contigs).

## 4. Discussion

Little has been known to date about the WGS-associated molecular epidemiology of MDR *E. coli* isolates resistant to carbapenems, aminoglycosides, and fluoroquinolones obtained from clinical settings in Vietnam. Treatment with a carbapenem was found to be an independent risk factor for CRE colonization [15]. In Vietnam, the isolation rate of MDR *E. coli* among MDR Gram-negative bacilli was lower than that of MDR *K. pneumoniae* [1], which is in accordance with this study (Table 1) and may explain the relatively few studies analyzing the molecular epidemiology of MDR *E. coli* using WGS in clinical setting of Vietnam. To our knowledge, the present study is the first to analyze the molecular epidemiology of aminoglycoside-resistant *E. coli* isolates obtained from clinical settings in Vietnam.

Based on several typing methods, including ST, O-type, H-type, plasmid type, and distribution of drug resistance genes, the MDR *E. coli* isolates from clinical setting of Vietnam were found to be highly heterogeneous. In contrast, MDR isolates of *Enterobacter cloacae* [16] and *Acinetobacter baumannii* [17] were more homogeneous, consisting primarily of the STs ST171 and CC2, respectively, and having similar drug resistance genes. These results suggested that the origins of MDR *E. coli* isolated from clinical settings in Vietnam differed markedly. It is possible that they have originated from raw meat and shellfish [2], retail meats [4, 6] and shrimp [5], hospital wastewater [6], backyard chicken farms [7], fecal sludge and soil [8], retail chicken carcasses [9], young dairy calves [10], urban rodents [5], and fish gut contents [11]. Because of their heterogeneity, the eradication of MDR *E. coli* from hospitals and environments is more challenging. In Southeast Asian and East Asian countries, antibiotics are easily available in community pharmacies [18–20]. Strict control of antibiotics use in such countries might contribute to exclude MDR *E. coli* in clinical setting of Vietnam.

In conclusion, limitations of this study include the relatively small number of isolates analyzed and the single-center nature of the study. Nevertheless, these results suggested that MDR *E. coli* isolates isolated from patients at a high-volume hospital in Vietnam are very heterogeneous regarding mechanisms to acquire resistance against antibiotics over the years. To control such heterogeneous bacterial population, adequate use of antibiotics could be the most effective way. Because MDR *E. coli* has been associated with higher in-hospital mortality rates, adequate measures are needed to prevent the spread of these bacteria. These findings are also the first to show fundamental aspects of MDR *E. coli* in a clinical setting in Vietnam.

## Figures and Tables

**Table 1:** Number of isolates and the percentage collected in this study.

Bacterial species	Number of isolate	%
*Acinetobacter baumannii*	704	62.1
*Klebsiella pneumoniae*	191	16.9
*Pseudomonas aeruginosa*	154	13.6
*Escherichia coli*	67	5.9
*Enterobacter cloacae*	17	1.5
Total	1133	

## Data Availability

All the WGS data were registered with the DNA Data Bank.

## Supplementary Materials

Supplementary Materials Table S1: drug resistance genes were analyzed using the CLC genomics workbench microbial genomics module (detection parameters: 99% identity and 50% length coverage with manual correction). Higher scores in the ratio of drug resistance genes among the 67 isolates are highlighted. Supplementary Table S2: PAIs detected in the isolates using PAIDB (90% identity and 50% length coverage with manual correction) (https://www.paidb.re.kr/about_paidb.php). Supplementary Table S3: distribution of virulence factors in isolates from Vietnam analyzed based on sequence data from VFDB (https://www.mgc.ac.cn/VFs/). Thresholds of 90% identity and 50% length fraction were used. Higher scores in the ratio of virulence factors are highlighted.
