# Supplementary material for: Molecular Epidemiology of Escherichia coli Resistant to Carbapenems, Fluoroquinolones, and Aminoglycosides Isolated from One of the Largest Hospitals in Vietnam in 2014–2019
Source: Can J Infect Dis Med Microbiol. 2024 Jan 31;2024:2711353. doi: 10.1155/2024/2711353 (PMC10849806; doi:10.1155/2024/2711353)
Supplement: Supplementary Materials — Table S1: drug resistance genes were analyzed using the CLC genomics workbench microbial genomics module (detection parameters: 99% identity and 50% length coverage with manual correction). Higher scores in the ratio of drug resistance genes among the 67 isolates are highlighted. Supplementary Table S2: PAIs detected in the isolates using PAIDB (90% identity and 50% length coverage with manual correction) (https://www.paidb.re.kr/about_paidb.php). Supplementary Table S3: distribution of virulence factors in isolates from Vietnam analyzed based on sequence data from VFDB (https://www.mgc.ac.cn/VFs/). Thresholds of 90% identity and 50% length fraction were used. Higher scores in the ratio of virulence factors are highlighted. [file 2711353.f1.zip › Table S2.docx]

Table S2. PAIs detected in the isolates using PAIDB (90 % identity and 50 % length coverage with manual correction) (http://www.paidb.re.kr/about_paidb.php).

| Gene | Number detected | Function | Insertion Site | GenBank Accession (Size) | Accession | Host Strain |
| --- | --- | --- | --- | --- | --- | --- |
| 04_HPI | 5 | Type IV secretion system, similar to FyuA/Psn receptor for siderophore yersiniabactin(fyuA) | tRNA-asnV | AY233333 (40.4kb, complete PAI) | AY233333 | *E. coli* ECOR31 |
| 14_ETT2 | 15 | Type III secretion system | tRNA-Gly | NC_002695_P1(27.5kb, complete PAI) | NC_002695 | *E. coli* O157:H7 Sakai |
| 16_Not_named | 15 | *tkt1* (transketolase) | *metE* and *ysgA* genes of the *E. coli* K12 genome | NC_008563_P5 (15.3kb, complete PAI) | NC_008563 | *E. coli* APEC O1 |
| 20_OI_57 | 5 | Non-LEE-encoded effector genes nleG2-3, nleG6-2, and nleG5-2 | - | NC_002655_P3 (9.2kb, complete PAI) | NC_002655 | *E. coli* O157:H7 EDL933 |
| 24_PAI_I_CFT073 | 1 | Alpha-hemolysin,  P-fimbriae, aerobactin | tRNA-pheV | NC_004431_P1(44.6kb, complete PAI) | NC_004431 | *E. coli* CFT073 |
| 26_PAI_II_APEC_O1 | 3 | - | tRNA-Asp | NC_008563_P1(29.7kb, complete PAI) | NC_008563 | *E. coli* APEC O1 |
| 31_PAI_IV_APEC_O1 | 20 | Yersiniabactic operon | tRNA-Asn | NC_008563_P3(77.7kb, complete PAI) | NC_008563 | *E. coli* APEC O1 |
